# *catena*-Poly[[di-μ-chlorido-dicopper(I)]bis­[μ-η^2^,σ^1^-4-(2-allyl-2*H*-tetra­zol-5-yl)pyridine]]

**DOI:** 10.1107/S1600536808017820

**Published:** 2008-06-19

**Authors:** Wei Wang

**Affiliations:** a Ordered Matter Science Research Center, Southeast University, Nanjing 210096, People’s Republic of China

## Abstract

The title polymer, [Cu_2_Cl_2_(C_9_H_9_N_5_)_2_]_*n*_, has been prepared by the solvothermal treatment of CuCl with 4-(2-allyl-2*H*-tetra­zol-5-yl)pyridine. The crystal structure shows that the title compound is a homometallic Cu^I^–olefin coordination polymer, in which the Cu_2_Cl_2_ nodes are bridged by two olefin ligands. The asymmetric unit contains one-half of the monomer, the complete monomer having twofold rotation symmetry. The coordination environment of Cu^I^ is slightly distorted tetra­hedral, with coordination sites being two μ_2_-Cl atoms, one pyridine N atom of an organic ligand and one allylic double bond of a symmetry-related ligand. Each organic mol­ecule behaves as a bidentate ligand, connecting two neighboring Cu_2_Cl_2_ dimers in the polymeric chain, which runs along [010].

## Related literature

For the solvothermal synthesis and for related structures, see: Ye *et al.* (2005 ▶, 2007 ▶). For related structures, see: Wang (2008*a*,*b*,*c*).
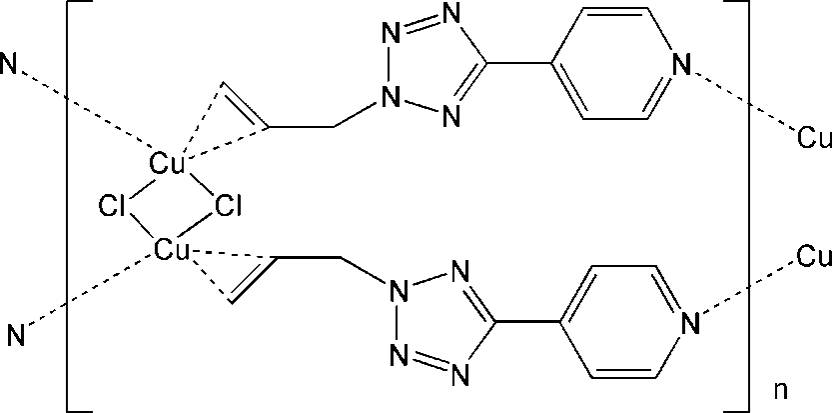


## Experimental

### 

#### Crystal data

[Cu_2_Cl_2_(C_9_H_9_N_5_)_2_]
*M* *_r_* = 286.21
Monoclinic, 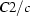

*a* = 17.270 (3) Å
*b* = 12.040 (2) Å
*c* = 13.064 (3) Å
β = 127.94 (3)°
*V* = 2142.3 (7) Å^3^
*Z* = 8
Mo *K*α radiation
μ = 2.27 mm^−1^
*T* = 293 (2) K
0.2 × 0.15 × 0.1 mm

#### Data collection

Rigaku Mercury2 diffractometer
Absorption correction: multi-scan (*CrystalClear*; Rigaku, 2005 ▶) *T*_min_ = 0.643, *T*_max_ = 0.800
10753 measured reflections
2451 independent reflections
1814 reflections with *I* > 2σ(*I*)
*R*_int_ = 0.059

#### Refinement

*R*[*F* ^2^ > 2σ(*F* ^2^)] = 0.041
*wR*(*F* ^2^) = 0.100
*S* = 1.06
2451 reflections
154 parameters
H-atom parameters constrained
Δρ_max_ = 0.33 e Å^−3^
Δρ_min_ = −0.39 e Å^−3^

### 

Data collection: *CrystalClear* (Rigaku, 2005 ▶); cell refinement: *CrystalClear*; data reduction: *CrystalClear*; program(s) used to solve structure: *SHELXS97* (Sheldrick, 2008 ▶); program(s) used to refine structure: *SHELXL97* (Sheldrick, 2008 ▶); molecular graphics: *PLATON* (Spek, 2003 ▶) and *XP* in *SHELXTL* (Sheldrick, 2008 ▶); software used to prepare material for publication: *SHELXTL*.

## Supplementary Material

Crystal structure: contains datablocks I, global. DOI: 10.1107/S1600536808017820/bh2171sup1.cif

Structure factors: contains datablocks I. DOI: 10.1107/S1600536808017820/bh2171Isup2.hkl

Additional supplementary materials:  crystallographic information; 3D view; checkCIF report

## Figures and Tables

**Fig. 1.**
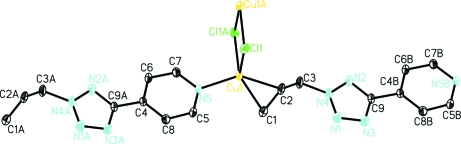
A view of a part of the title polymer, with atomic numbering scheme. Displacement ellipsoids were drawn at the 30% probability level. Symmetry codes: (A) x, y - 1, z; (B) x, y + 1, z.

**Fig. 2.**
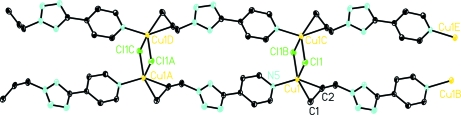
The one-dimensional chain structure of the title compound.

## supplementary crystallographic information

### Comment

Under hydrothermal or solvothermal conditions, some interesting reactions and compounds can be obtained, while these products could not be synthesized using conventional solution techniques. In sealed tubes, unstable Cu^I^ salts can exist under reduced pressure, and then interesting Cu^I^ coordination compounds can be obtained. The title compound is obtained through solvothermal treatment of CuCl and 4-(2-allyl-2*H*-tetrazol-5-yl)pyridine in methanol solvent at 348 K. Colourless block crystals suitable for X-ray diffractions have been isolated.

The Cu^I^ ion is coordinated to two olefin ligands and two bridging Cl atoms in a tetrahedral environment (Fig. 1). Two olefin ligands related by a twofold axis link the neighbouring Cu_2_Cl_2_ dimers to form an homometallic Cu^I^ olefin coordination polymer, developing along the [010] axis, with the Cu_2_Cl_2_ dimers acting as nodes. The allyl groups coordinate to Cu^I^ centers through N atoms of pyridine rings and double bonds of allyl groups. Unfortunately, the N atoms of tetrazole rings fail to coordinate Cu^I^ ions (Fig. 2).

### Experimental

A mixture of 4-(2-allyl-2*H*-tetrazol-5-yl)pyridine (20 mg, 0.2 mmol), CuCl (36 mg, 0.4 mmol), and methanol (2 ml) sealed in a glass tube were maintained at 348 K. Crystals suitable for X-ray analysis were obtained after 5 days.

### Refinement

All H atoms were placed geometrically and treated as riding with C—H = 0.93 (aromatic), 0.97 (methylene) or 0.96 Å (methyl), with *U*_iso_(H) = 1.2*U*_eq_(Caromatic, Cmethylene) or *U*_iso_(H) = 1.5*U*_eq_(Cmethyl).

### Crystal data

**Table:**

[Cu_2_Cl_2_(C_9_H_9_N_5_)_2_]	*F*(000) = 1152
*M**_r_* = 286.21	*D*_x_ = 1.775 Mg m^−^^3^
Monoclinic, *C*2/*c*	Mo *K*α radiation, λ = 0.71073 Å
Hall symbol: -C 2yc	Cell parameters from 9724 reflections
*a* = 17.270 (3) Å	θ = 3.2–28.8°
*b* = 12.040 (2) Å	µ = 2.27 mm^−^^1^
*c* = 13.064 (3) Å	*T* = 293 K
β = 127.94 (3)°	Block, colourless
*V* = 2142.3 (7) Å^3^	0.2 × 0.15 × 0.1 mm
*Z* = 8	

### Data collection

**Table:**

Rigaku Mercury2 diffractometer	2451 independent reflections
Radiation source: fine-focus sealed tube	1814 reflections with *I* > 2σ(*I*)
graphite	*R*_int_ = 0.059
Detector resolution: 13.6612 pixels mm^-1^	θ_max_ = 27.5°, θ_min_ = 3.2°
CCD_Profile_fitting scans	*h* = −22→22
Absorption correction: multi-scan (*CrystalClear*; Rigaku, 2005)	*k* = −15→15
*T*_min_ = 0.643, *T*_max_ = 0.800	*l* = −16→16
10753 measured reflections	

### Refinement

**Table:**

Refinement on *F*^2^	Primary atom site location: structure-invariant direct methods
Least-squares matrix: full	Secondary atom site location: difference Fourier map
*R*[*F*^2^ > 2σ(*F*^2^)] = 0.041	Hydrogen site location: inferred from neighbouring sites
*wR*(*F*^2^) = 0.100	H-atom parameters constrained
*S* = 1.06	*w* = 1/[σ^2^(*F*_o_^2^) + (0.0422*P*)^2^ + 0.6044*P*] where *P* = (*F*_o_^2^ + 2*F*_c_^2^)/3
2451 reflections	(Δ/σ)_max_ = 0.001
154 parameters	Δρ_max_ = 0.33 e Å^−^^3^
0 restraints	Δρ_min_ = −0.39 e Å^−^^3^

### Fractional atomic coordinates and isotropic or equivalent isotropic displacement parameters (Å^2^)

**Table:**

	*x*	*y*	*z*	*U*_iso_*/*U*_eq_	
Cu1	0.39314 (3)	0.49203 (3)	0.59806 (4)	0.03726 (16)	
Cl1	0.57394 (6)	0.49142 (6)	0.69868 (8)	0.0343 (2)	
N1	0.3855 (2)	0.8479 (2)	0.3785 (3)	0.0460 (7)	
N2	0.40548 (19)	0.91092 (19)	0.5524 (3)	0.0362 (6)	
N3	0.3659 (2)	0.9552 (2)	0.3613 (3)	0.0435 (7)	
N4	0.40899 (19)	0.82409 (19)	0.4924 (3)	0.0345 (6)	
N5	0.36588 (18)	0.33219 (19)	0.5431 (2)	0.0304 (6)	
C1	0.2946 (2)	0.5831 (2)	0.4346 (3)	0.0388 (8)	
H1A	0.2411	0.5472	0.4259	0.068 (12)*	
H1C	0.3062	0.5683	0.3729	0.052 (11)*	
C2	0.3534 (2)	0.6549 (2)	0.5330 (3)	0.0350 (7)	
H2A	0.3419	0.6698	0.5948	0.089 (15)*	
C3	0.4367 (2)	0.7121 (2)	0.5475 (3)	0.0402 (8)	
H3A	0.4545	0.6695	0.5027	0.030 (8)*	
H3B	0.4928	0.7163	0.6374	0.052 (11)*	
C4	0.3685 (2)	0.1086 (2)	0.4905 (3)	0.0273 (6)	
C5	0.3489 (2)	0.2998 (2)	0.4331 (3)	0.0345 (7)	
H5A	0.3356	0.3559	0.3720	0.041 (9)*	
C6	0.3829 (2)	0.1409 (2)	0.6025 (3)	0.0321 (7)	
H6A	0.3937	0.0860	0.6633	0.047 (10)*	
C7	0.3811 (2)	0.2523 (2)	0.6256 (3)	0.0317 (7)	
H7A	0.3911	0.2741	0.7037	0.033 (8)*	
C8	0.3495 (2)	0.1902 (2)	0.4032 (3)	0.0346 (7)	
H8A	0.3371	0.1704	0.3231	0.054 (11)*	
C9	0.3785 (2)	0.9917 (2)	0.4676 (3)	0.0301 (7)	

### Atomic displacement parameters (Å^2^)

**Table:**

	*U*^11^	*U*^22^	*U*^33^	*U*^12^	*U*^13^	*U*^23^
Cu1	0.0528 (3)	0.0168 (2)	0.0349 (2)	−0.00049 (16)	0.0233 (2)	−0.00040 (15)
Cl1	0.0435 (4)	0.0304 (4)	0.0372 (4)	0.0055 (3)	0.0290 (4)	0.0058 (3)
N1	0.069 (2)	0.0269 (14)	0.0416 (16)	0.0058 (13)	0.0335 (16)	−0.0005 (12)
N2	0.0465 (15)	0.0205 (12)	0.0401 (15)	−0.0013 (11)	0.0259 (13)	0.0008 (11)
N3	0.067 (2)	0.0263 (14)	0.0390 (16)	0.0078 (13)	0.0332 (16)	0.0024 (12)
N4	0.0431 (16)	0.0165 (12)	0.0435 (16)	0.0016 (10)	0.0265 (14)	−0.0001 (11)
N5	0.0371 (14)	0.0179 (12)	0.0321 (13)	−0.0020 (10)	0.0191 (12)	−0.0013 (10)
C1	0.0421 (19)	0.0278 (16)	0.0394 (18)	0.0039 (14)	0.0215 (16)	0.0070 (14)
C2	0.052 (2)	0.0180 (14)	0.0432 (19)	0.0075 (13)	0.0337 (18)	0.0074 (13)
C3	0.043 (2)	0.0164 (14)	0.051 (2)	0.0045 (13)	0.0234 (18)	0.0053 (14)
C4	0.0293 (15)	0.0182 (14)	0.0291 (15)	−0.0021 (11)	0.0153 (13)	−0.0002 (11)
C5	0.0465 (19)	0.0197 (14)	0.0325 (18)	−0.0001 (13)	0.0218 (16)	0.0043 (12)
C6	0.0415 (18)	0.0191 (14)	0.0348 (17)	−0.0026 (12)	0.0229 (15)	0.0023 (13)
C7	0.0423 (18)	0.0228 (15)	0.0328 (17)	−0.0037 (12)	0.0245 (16)	−0.0014 (12)
C8	0.0468 (19)	0.0257 (15)	0.0319 (17)	−0.0024 (13)	0.0246 (15)	−0.0009 (13)
C9	0.0357 (16)	0.0170 (14)	0.0347 (16)	−0.0027 (12)	0.0202 (14)	−0.0002 (12)

### Geometric parameters (Å, °)

**Table:**

Cu1—N5	2.006 (2)	C1—H1C	0.9600
Cu1—C1	2.047 (3)	C2—C3	1.497 (4)
Cu1—C2	2.079 (3)	C2—H2A	0.9599
Cu1—Cl1^i^	2.3491 (11)	C3—H3A	0.9598
Cu1—Cl1	2.5358 (12)	C3—H3B	0.9599
Cl1—Cu1^i^	2.3491 (11)	C4—C8	1.384 (4)
N1—N4	1.310 (4)	C4—C6	1.381 (4)
N1—N3	1.319 (4)	C4—C9^ii^	1.471 (4)
N2—C9	1.325 (4)	C5—C8	1.378 (4)
N2—N4	1.330 (3)	C5—H5A	0.9600
N3—C9	1.341 (4)	C6—C7	1.379 (4)
N4—C3	1.464 (3)	C6—H6A	0.9599
N5—C5	1.336 (4)	C7—H7A	0.9600
N5—C7	1.345 (4)	C8—H8A	0.9600
C1—C2	1.351 (4)	C9—C4^iii^	1.471 (4)
C1—H1A	0.9600		
			
N5—Cu1—C1	106.18 (11)	C3—C2—Cu1	109.4 (2)
N5—Cu1—C2	144.35 (12)	C1—C2—H2A	119.7
C1—Cu1—C2	38.23 (12)	C3—C2—H2A	119.1
N5—Cu1—Cl1^i^	104.01 (8)	Cu1—C2—H2A	91.1
C1—Cu1—Cl1^i^	130.46 (11)	N4—C3—C2	111.3 (3)
C2—Cu1—Cl1^i^	104.77 (10)	N4—C3—H3A	108.8
N5—Cu1—Cl1	97.23 (8)	C2—C3—H3A	108.7
C1—Cu1—Cl1	120.78 (11)	N4—C3—H3B	109.4
C2—Cu1—Cl1	101.90 (10)	C2—C3—H3B	110.4
Cl1^i^—Cu1—Cl1	92.81 (5)	H3A—C3—H3B	108.2
Cu1^i^—Cl1—Cu1	87.19 (5)	C8—C4—C6	118.1 (3)
N4—N1—N3	106.1 (3)	C8—C4—C9^ii^	120.6 (3)
C9—N2—N4	101.8 (2)	C6—C4—C9^ii^	121.2 (3)
N1—N3—C9	106.4 (3)	N5—C5—C8	123.4 (3)
N1—N4—N2	113.7 (2)	N5—C5—H5A	118.0
N1—N4—C3	122.7 (3)	C8—C5—H5A	118.6
N2—N4—C3	123.6 (3)	C7—C6—C4	119.5 (3)
C5—N5—C7	117.3 (2)	C7—C6—H6A	120.5
C5—N5—Cu1	120.90 (19)	C4—C6—H6A	119.9
C7—N5—Cu1	120.8 (2)	N5—C7—C6	122.6 (3)
C2—C1—Cu1	72.17 (18)	N5—C7—H7A	118.4
C2—C1—H1A	120.4	C6—C7—H7A	119.0
Cu1—C1—H1A	90.3	C4—C8—C5	119.0 (3)
C2—C1—H1C	119.6	C4—C8—H8A	120.2
Cu1—C1—H1C	107.9	C5—C8—H8A	120.8
H1A—C1—H1C	120.0	N2—C9—N3	112.0 (3)
C1—C2—C3	121.1 (3)	N2—C9—C4^iii^	123.9 (3)
C1—C2—Cu1	69.60 (17)	N3—C9—C4^iii^	124.0 (3)

Symmetry codes: (i) −*x*+1, *y*, −*z*+3/2; (ii) *x*, *y*−1, *z*; (iii) *x*, *y*+1, *z*.

### Hydrogen-bond geometry (Å, °)

**Table:**

*D*—H···*A*	*D*—H	H···*A*	*D*···*A*	*D*—H···*A*
C7—H7A···Cl1^i^	0.96	2.81	3.459 (3)	126.

Symmetry codes: (i) −*x*+1, *y*, −*z*+3/2.
